# Templated growth of PFO-DBT nanorod bundles by spin coating: effect of spin coating rate on the morphological, structural, and optical properties

**DOI:** 10.1186/1556-276X-9-225

**Published:** 2014-05-08

**Authors:** Muhamad Saipul Fakir, Azzuliani Supangat, Khaulah Sulaiman

**Affiliations:** 1 Low Dimensional Materials Research Centre, Department of Physics, University of Malaya, Kuala Lumpur 50603, Malaysia

**Keywords:** PFO-DBT, Nanorods, Spin coating rate, Porous alumina template

## Abstract

In this study, the spin coating of template-assisted method is used to synthesize poly[2,7-(9,9-dioctylfluorene)-alt-4,7-bis(thiophen-2-yl)benzo-2,1,3-thiadiazole] (PFO-DBT) nanorod bundles. The morphological, structural, and optical properties of PFO-DBT nanorod bundles are enhanced by varying the spin coating rate (100, 500, and 1,000 rpm) of the common spin coater. The denser morphological distributions of PFO-DBT nanorod bundles are favorably yielded at the low spin coating rate of 100 rpm, while at high spin coating rate, it is shown otherwise. The auspicious morphologies of highly dense PFO-DBT nanorod bundles are supported by the augmented absorption and photoluminescence.

## Background

In recent years, poly[2,7-(9,9-dioctylfluorene)-alt-4,7-bis(thiophen-2-yl)benzo-2,1,3-thiadiazole] (PFO-DBT) has attracted numerous attention due to its exceptional optical properties. Applications in electronic devices such as solar cells and light-emitting diodes have elevated PFO-DBT thin films to be one of the most promising materials [1-6] in accordance with its capability in absorbing and emitting light effectively. In solar cell application, the harvested light at longer wavelength of PFO-DBT thin film matches with solar radiation [3,4]. Although, PFO-DBT films and nanostructures have the same properties in absorption, PFO-DBT nanostructures can exhibit more surface area which can enhance light absorption. Nanostructured materials have been proven to extremely exhibit large surface area and substantial light absorption intensity [7-9]. Considerations on nanostructured formation have been prioritized due to the superior morphological and optical properties [8,10-13]. Introducing nanostructure would enhance the light absorption intensity, and the low absorption issue of PFO-DBT thin film can be overcome. Therefore, the fabrication of PFO-DBT nanostructures such as nanotubes, nanorods, and other novel nanostructures formation is rather essential and pragmatic.

One of the mutual approaches in fabricating the nanostructures is template-assisted method. Template-assisted method has been generally used to produce the unique nanostructured materials [8,10,14-16]. By using the template, various shapes and properties of nanostructures can be formed. The dimension of nanostructures can be controlled by varying either the thickness or the diameter of porous template. However, the formation in zero-, one-, two-or three-dimensional nanostructures can be controlled by applying various infiltration techniques during the deposition of polymer solution into porous alumina template [10,12-16]. Among the infiltration techniques are wetting-, vacuum-, and spin-based techniques. A spin-based technique of spin coater is potentially used as a lucrative and superficial fabrication tool.

Spin coating of solution into the porous template can possibly enhance the infiltration. On the planar substrate, the thickness of macroporous polymers can be easily tuned by varying the spin coating rate [13], in which the different behaviors of materials during spin coating have to be the main influence. Commonsensically, the behavior of a polymer solution would probably be affected by the spin coating rate during the deposition onto the porous substrate of alumina template due to the changes of surface energy [16]. Modification on the morphological, structural, and optical properties of PFO-DBT nanostructures that were synthesized by varying the spin coating rate has not been widely studied. Therefore, it is noteworthy to study the effect of the spin coating rate on the morphological, structural, and optical properties of PFO-DBT nanostructures. This work is crucial since it provides an alternative method to utilize the facile fabrication technique.

## Methods

The commercially existing copolymer of PFO-DBT from Lum-Tec (Mentor, OH, USA) was utilized without further purification. A 5-mg/ml solution concentration of PFO-DBT was dissolved in chloroform. Commercially available porous alumina template from Whatman Anodisc Inorganic Membrane (Sigma-Aldrich, St. Louis, MO, USA) with nominal pore diameter of 20 nm and a thickness of 60 μm was cleaned by sonicating it in water and acetone for 10 min prior to the deposition of PFO-DBT solution. The PFO-DBT solution was dropped onto the porous alumina template prior to the spin coating process. The spin coating rate was varied to 100, 500, and 1000 rpm at a constant spin time of 30 s, by using a standard spin coater model WS-650MZ-23NPP (Laurell Technologies Corp., North Wales, PA, USA). In order to dissolve the template, 3 M of sodium hydroxide (NaOH) was used, leaving the PFO-DBT nanorods. The PFO-DBT nanorods were purified in deionized water prior to its characterization. The characterizations of PFO-DBT nanorods were performed using a field emission scanning electron microscope (FESEM) (Quanta FEG 450, Beijing, China), transmission electron microscope (TEM) (Tecnai G2 FEI, Tokyo, Japan), X-ray diffraction spectroscope (Siemens, Selangor, Malaysia), UV-vis spectroscope (Jasco V-750, Tokyo, Japan), and photoluminescence spectroscope (Renishaw).

## Results and discussion

### Morphological properties

**Figure 1:**
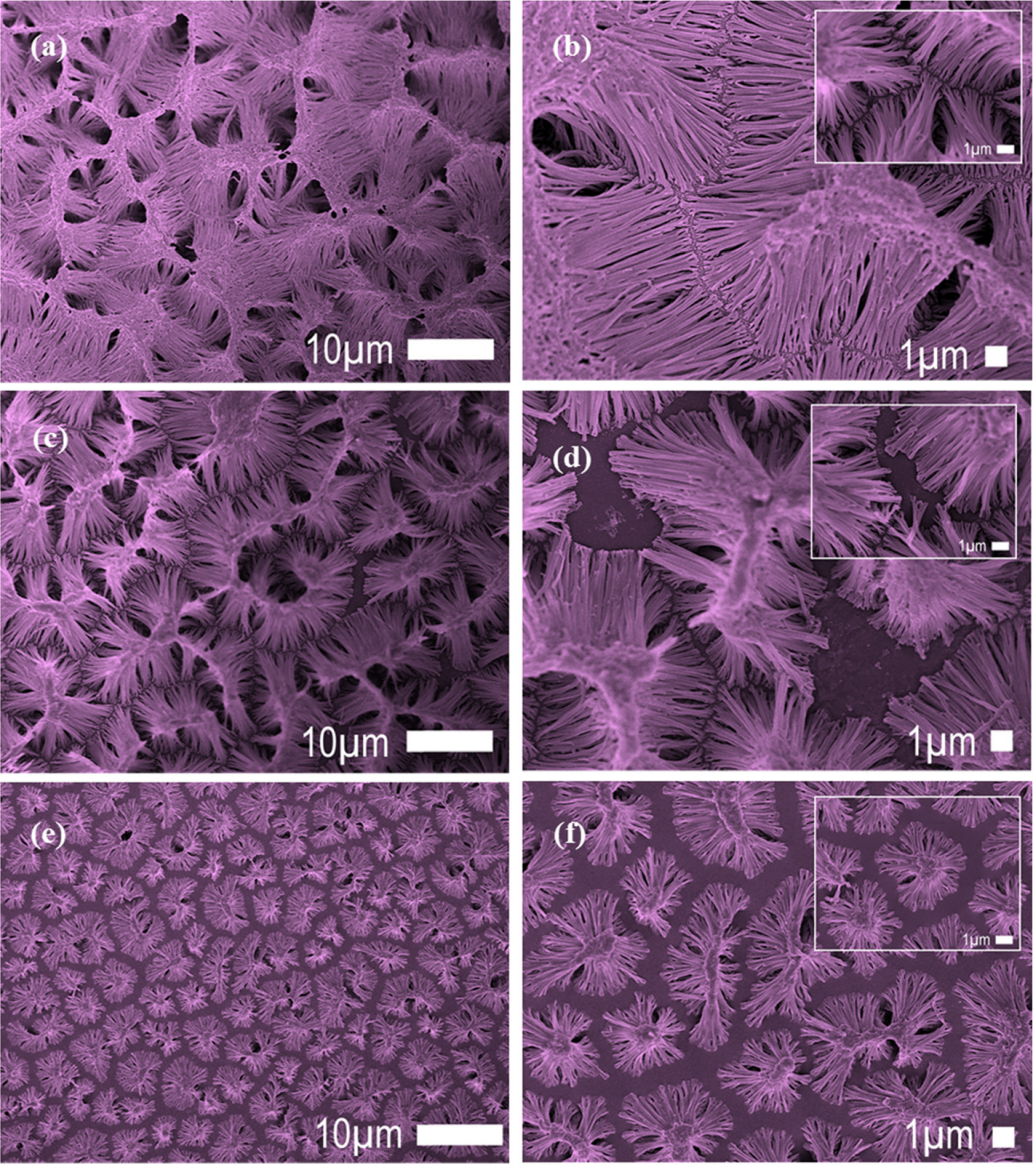
**FESEM images of PFO-DBT nanorod bundles with different spin coating rates.** FESEM images of PFO-DBT nanorod bundles with different spin coating rates of **(a)** 100 rpm at lower magnification, **(b)** 100 rpm at higher magnification, **(c)** 500 rpm at lower magnification, **(d)** 500 rpm at higher magnification, **(e)** 1,000 rpm at lower magnification, and **(f)** 1,000 rpm at higher magnification. The insets show enlarged images (scale bar, 1 μm).

A common practice in producing nanostructured materials via template-assisted method is by drop casting the solution on the template. However, the drop casting alone without the assistance of a spin coating technique would not efficiently allow the solution to infiltrate into the template. Infiltration of PFO-DBT solution into the cavity of an alumina template can be done by varying the spin coating rate. The FESEM images of the PFO-DBT nanorod bundles are shown in Figure 1a,b,c,d,e,f. Distinct morphological distribution of the PFO-DBT nanorod bundles are depicted by the different spin coating rates (100, 500, and 1000 rpm). It is expected that by varying the spin coating rate from low (100 rpm), intermediate (500 rpm), and high (1000 rpm), dissimilar morphological distributions will result. At all spin coating rates, the PFO-DBT nanorod bundles are seen to ensemble, however, with different densifications of morphological distribution.

**Figure 2:**
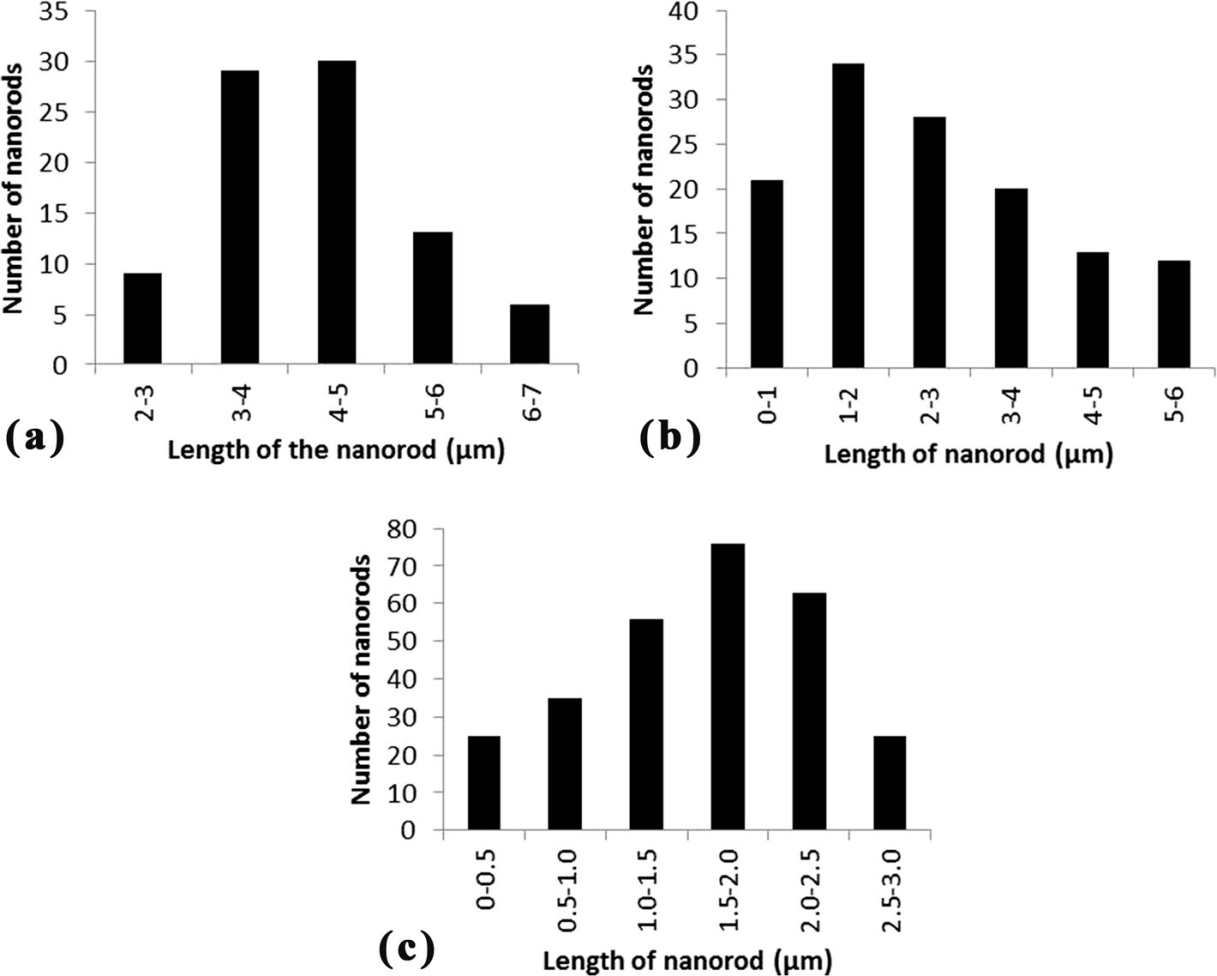
**Number of nanorods as a function of length in 15 μm × 15 μm area.** Spin coating rate at **(a)** 100 rpm, **(b)** 500 rpm, and **(c)** 1000 rpm.

**Figure 3:**
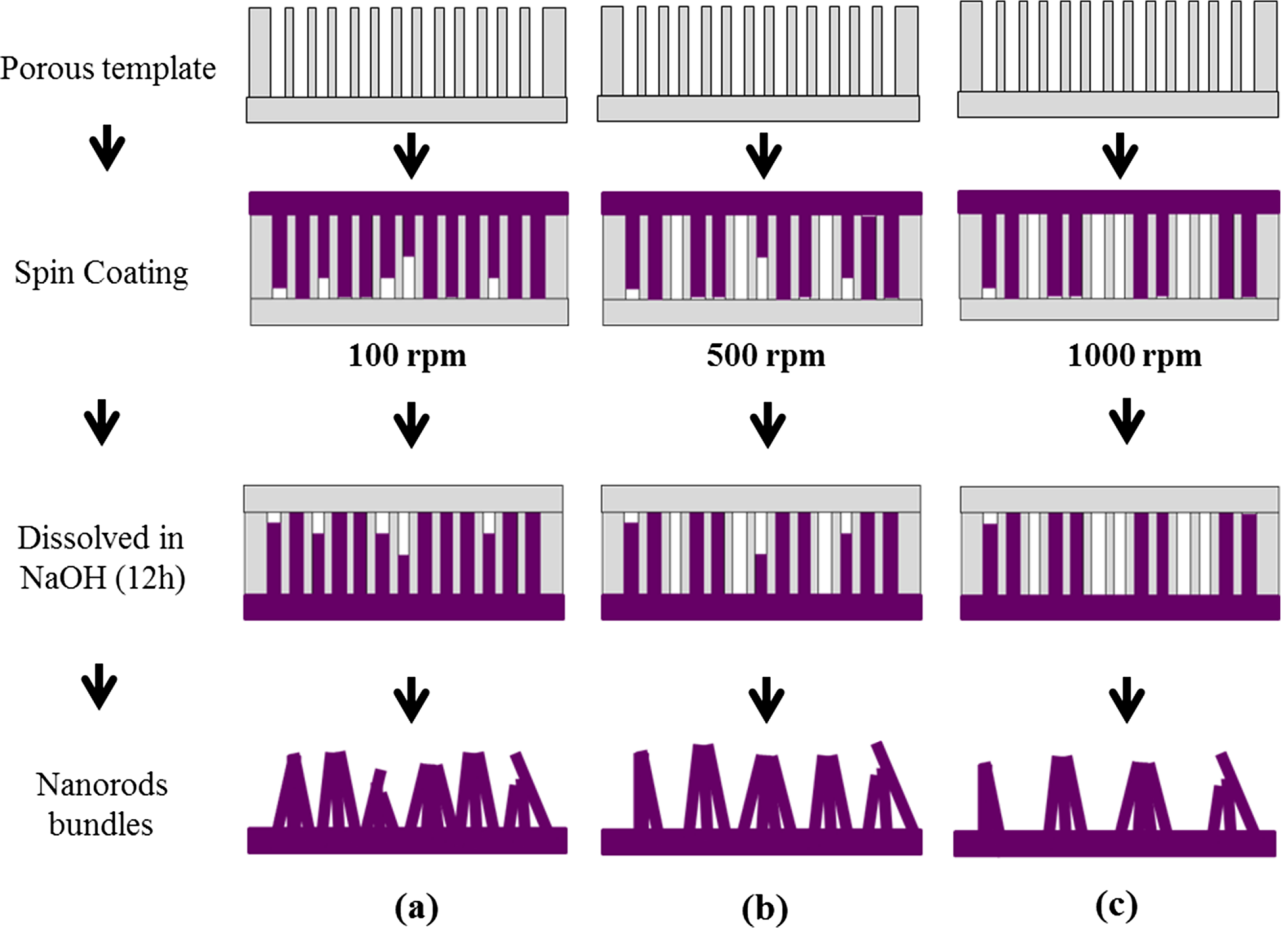
**Schematic illustrations of the PFO-DBT nanorod bundles (side view).** PFO-DBT nanorod bundles synthesized at different spin coating rates of **(a)** 100 rpm, **(b)** 500 rpm, and **(c)** 1000 rpm.

At the low spin coating rate of 100 rpm, the denser PFO-DBT nanorod bundles are synthesized. Looking at the top of the bundles, the tips of the nanorods are tending to join with one another which could be due to the van der Waals force interaction. Apart of that, the high aspect ratio of the PFO-DBT nanorods obtained at low spin coating rate can be one of the contributions as well. However, the main contribution to the distinct morphological distribution is merely the different behaviors exhibited by PFO-DBT during the spin coating. The smallest diameter recorded at 100, 500, and 1,000 rpm is 370, 200, and 100 nm, respectively. An analysis of nanorods' length is depicted in Figure 2 by bar graphs. For 100, 500, and 1,000 rpm, the average length is 3 to 5 μm, 1 to 3 μm, and 1.5 to 2.5 μm, respectively. Although the length is quite uniform, the nanorods' length is still affected by the spin coating rate. Figure 3a,b,c shows the proposed diagrams of the PFO-DBT nanorod bundles synthesized at different spin coating rates from the side view. As reported elsewhere, the resulting polymer films are highly dependent on the characteristics of spin coating [17]. Thus, it is sensible to predict that the structure formation of resulting films can be straightforwardly controlled by altering the spin coating rate. The mechanism of the controlled PFO-DBT nanorod bundles is affected by the phase transitions of the spin-coated polymer solution. Sensibly, the infiltration properties between the static and vibrate polymer solution holds an enormous transformation. The most remarkable attribute of spin coating rate is the occurrence of enhanced infiltration. The PFO-DBT nanorods have undergone three phase transitions: from less infiltration (1,000 rpm) to high infiltration (100 rpm), in which medium infiltration can be achieved at 500 rpm. At low spin rate, the low centrifugal force allows the polymer enough time from its starting position to infiltrate all of the surrounding porous gaps.

**Figure 4:**
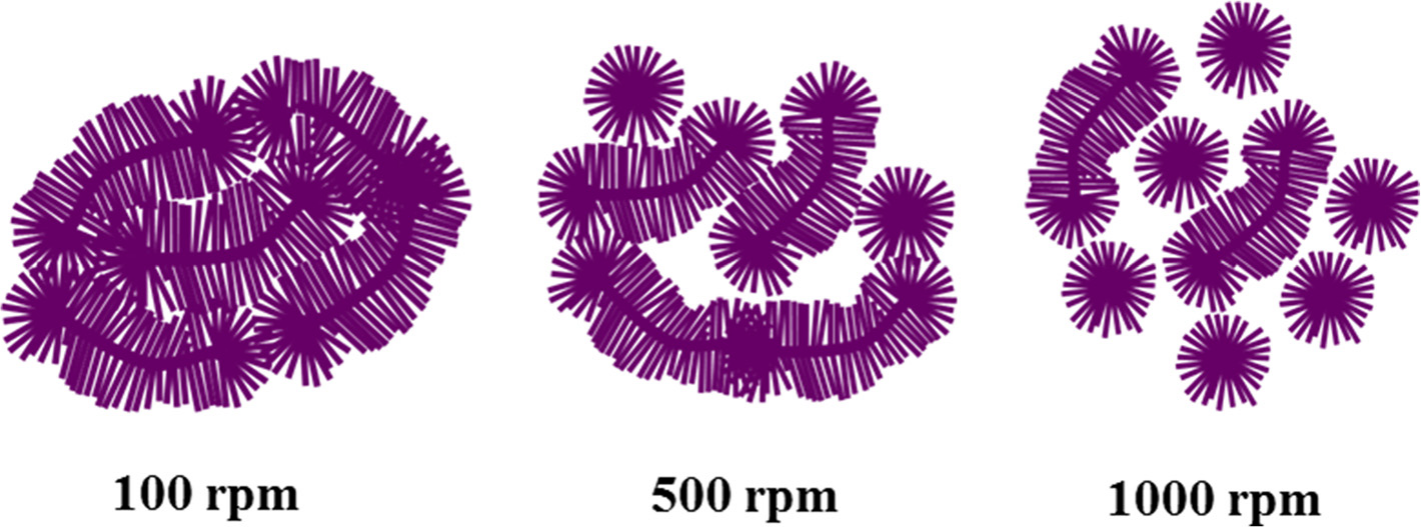
Schematic illustrations of the PFO-DBT nanorod bundles (top view).

Depending on the applications, the morphological distributions of the PFO-DBT nanorods can be simply tuned via the spin coating of template-assisted method. Further corroboration on the effect of spin coating rate can be confirmed by the ability of the PFO-DBT solution to occupy the cavity of the template. At the intermediate spin coating rate (500 rpm), the gaps between the nanorod bundles started to form. The formation of these gaps may be due to the infirmity of PFO-DBT solution to occupy the cavity. In other words, the gap corresponded to the unoccupied cavity that will be dissolved with NaOH. Auxiliary increase of centrifugal force in spin coating rate will create an intense gap between the nanorod bundles which is identical to the scattered islands. Rapid evaporation of the PFO-DBT solution at 1,000 rpm has caused the formation of scattered islands. The top view images of the PFO-DBT nanorod bundles are illustrated in Figure 4. These diagrams corresponded to the FESEM images taken from the top view (see Figure 1). Highly dense PFO-DBT nanorods can be obtained from the low spin coating rate of 100 rpm.

**Figure 5:**
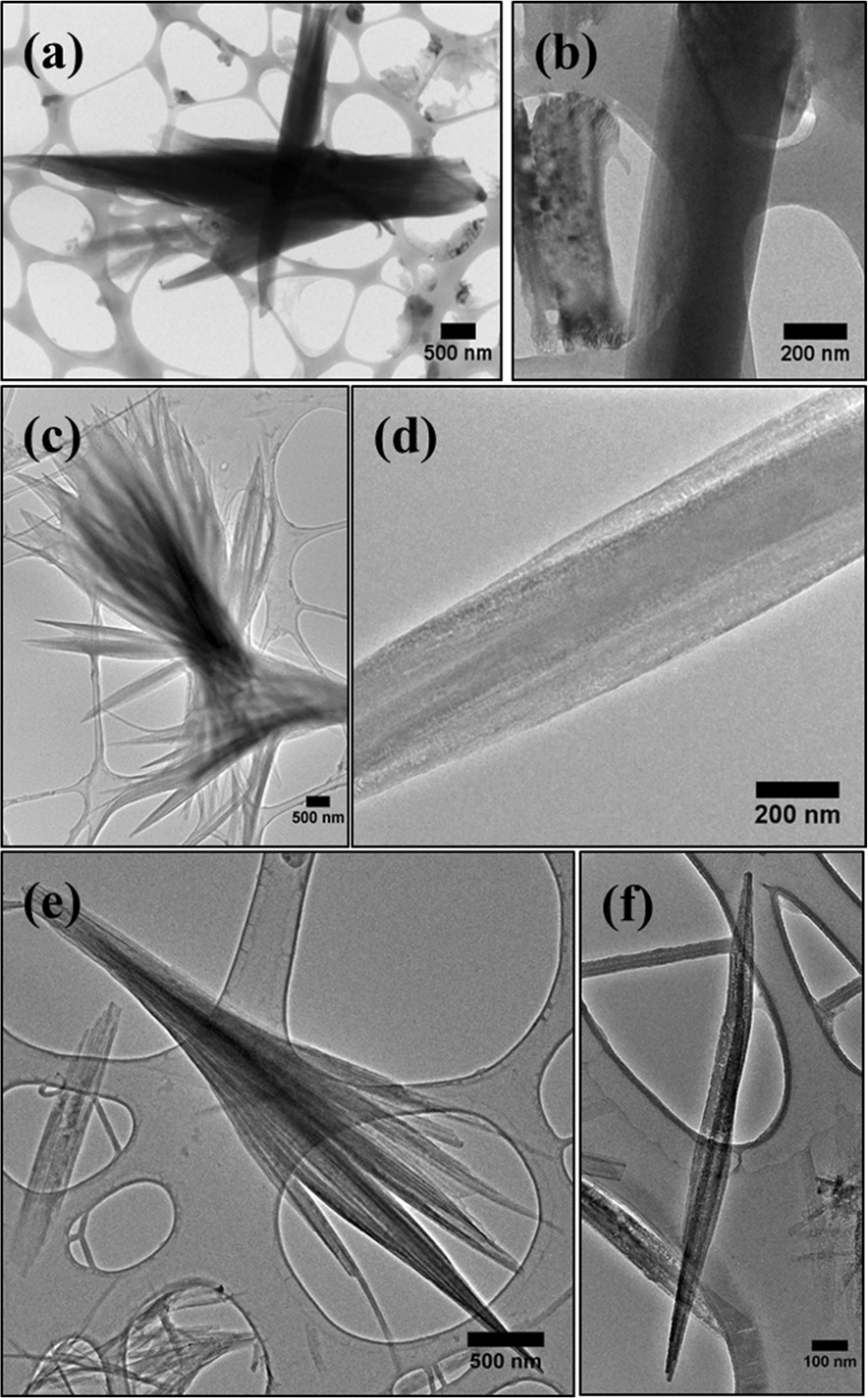
**TEM images of the PFO-DBT nanorod bundles with different spin coating rates.** TEM images of PFO-DBT nanorod bundles with different spin coating rates of **(a)** 100 rpm at lower magnification, **(b)** 100 rpm at higher magnification, **(c)** 500 rpm at lower magnification, **(d)** 500 rpm at higher magnification, **(e)** 1,000 rpm at lower magnification, and **(f)** 1000 rpm at higher magnification.

The morphologies of the PFO-DBT nanorod bundles are further supported by the TEM images (Figure 5a,b,c,d,e,f). As expected, distinct morphological distributions as an ensemble are recorded from the different spin coating rates. The highly dense PFO-DBT nanorod bundles are obtained at 100 rpm. At this spin coating rate, the greater numbers of nanorods are produced which could cause the bundles to agglomerate. Agglomeration of bundles in TEM images taken from the different spin coating rates agreed with the FESEM images; however, rigorous TEM preparation has initiated the broken and defected nanorods. An individual TEM image has confirmed that the nanorods are the sort of nanostructures obtained in this synthesis. It can be seen from the formation of solid structure without the composition of tubes (wall thickness).

### Structural properties

**Figure 6:**
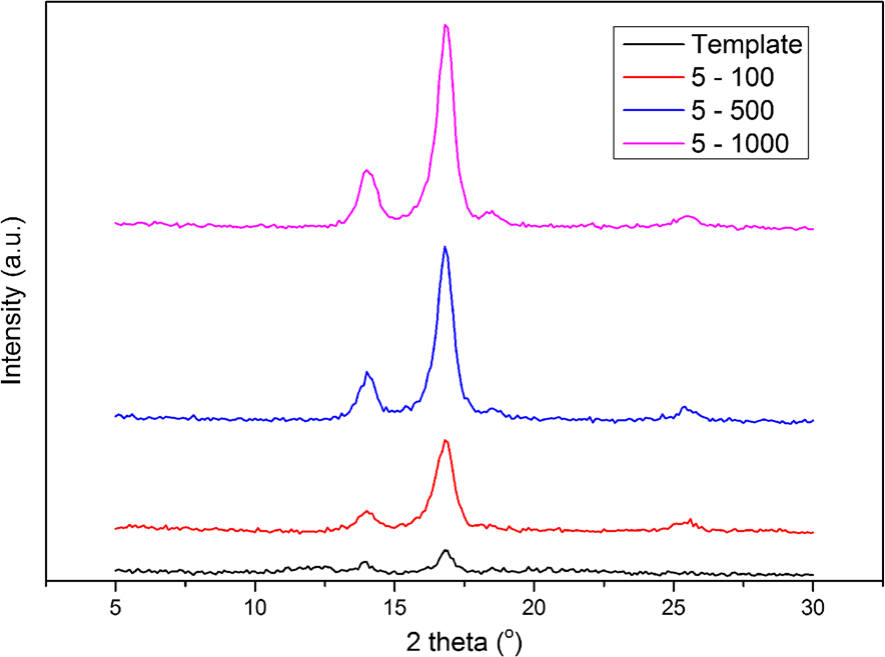
**X-ray diffraction (XRD) patterns of template and PFO-DBT nanorods.** The nanorods were grown inside the template of different spin coating rates.

The structural properties of the PFO-DBT nanorods are investigated by XRD. Figure 6 shows the XRD patterns of template and PFO-DBT nanorods grown inside the template of different spin coating rates. Diffraction peaks of porous alumina template are exhibited at 13.3° and 16.8°. All the PFO-DBT nanorods that grown inside the template have an additional diffraction peak at 25.2°. The additional diffraction peak is nearly similar to that reported by Wang et al. [1]. Sharper diffraction peaks are observed from the diffraction peaks of the PFO-DBT nanorods which indicate a semi-crystalline polymer. The PFO-DBT nanorod is confined inside the cavity of the template which then alters its molecular structure to a more aligned and elongated chain segment [11,12]. The crystallite size of the PFO-DBT nanorods can be verified using the Scherrer equation as shown in Equation 1:

(1) $$L=\frac{\mathrm{K\lambda}}{\beta \cos\theta }$$

From this equation, *L* is the mean crystallite size, *K* is the Scherrer constant with value 0.94, *λ* = 1.542 Å is the X-ray source wavelength, and *β* is the FWHM value. The PFO-DBT crystallite size is around 20 to 30 nm. The PFO-DBT nanorods that have been deposited inside the porous template exhibited a semi-crystalline polymer with enhanced polymer chain due to the restricted intrusion into the cavities.

### Optical properties

**Figure 7:**
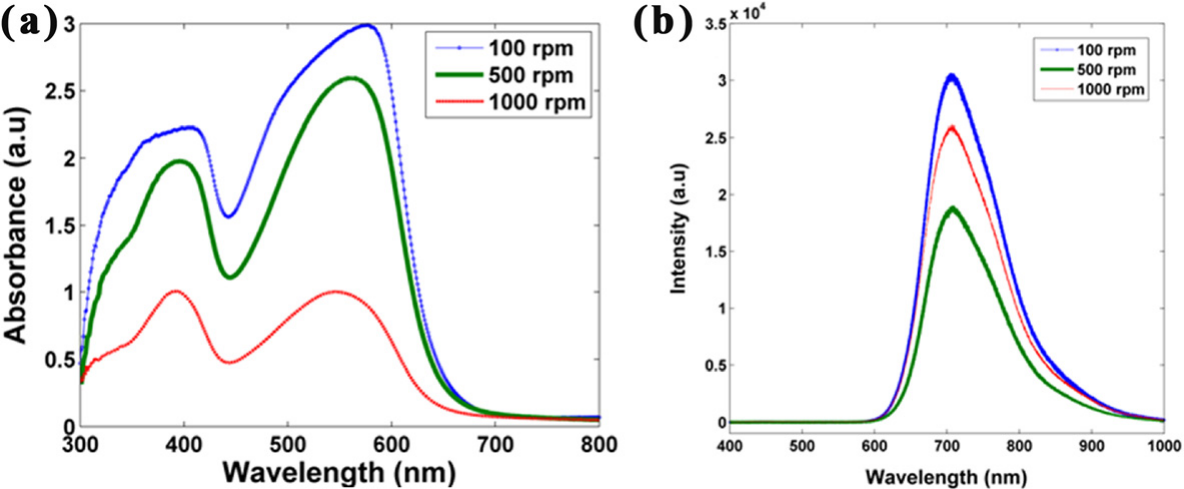
**Optical spectra of the PFO-DBT nanorod bundles. (a)** UV-vis absorption spectra. **(b)** Photoluminescence spectra.

The absorption spectra of the PFO-DBT nanorod bundles with different spin coating rates are shown in Figure 7a. These spectra portray two absorption peaks mainly assigned to PFO segments (short wavelength) and DBT units (long wavelength). The absorption band of the PFO-DBT thin film has been reported to locate at 388 nm (short wavelength) and 555 nm (long wavelength) [2,4]. Enhancement on the PFO-DBT's optical properties can be realized with the low spin coating rate of 100 rpm. With the denser distribution of the PFO-DBT nanorod bundles, the absorption band at short wavelength and long wavelength is shifted to 408 and 577 nm, respectively. The absorption peak of the PFO-DBT nanorod bundles at short wavelength is redshifted at approximately 20 nm compared to that of the PFO-DBT thin film reported by Wang et al. [4]. The peak at short wavelength corresponds to the transition of π- π* at fluorene units [4], which indicates that the strong π-π* transition has occurred via the denser PFO-DBT nanorod bundles. At the long wavelength, the PFO-DBT nanorod bundles that were obtained at the low spin coating rate of 100 rpm were recorded to have an absorption band at 577 nm which was assigned for the DBT units [3]. The maximum peak of 577 nm yields the higher intensity which indicates that the absorption of dioctylfluorene moieties is assisted by the thiophene [18]. The redshift of the absorption peaks is correlated with the morphological distribution of PFO-DBT nanorod bundles. It can be postulated that the highly dense nanorod bundles with close pack arrangement would give a better conjugation length and chain segment. Such improvement in conjugation length can be utilized to enhance the photovoltaic properties of polymeric solar cell. The morphological distribution of the PFO-DBT nanorod bundles has a significant contribution to their optical properties. The optical properties of polymer can be easily tuned by varying the spin coating rate, which indeed gives the different morphological distributions. This postulation can be further proven by the UV-vis spectra of the PFO-DBT nanorod bundles prepared at 500 and 1,000 rpm. With the implementation of spin coating rates of 500 and 1000 rpm, the absorption band at long wavelength are blueshifted at about 12 and 32 nm, respectively.

The photoluminescence (PL) spectra of the PFO-DBT nanorod bundles synthesized at different spin coating rates are shown in Figure 7b. The emission of the fluorene segment which normally lied between 400 and 550 nm [2,5,6] is not recorded by all of the spectra. It indicates that the fluorene unit has been completely quenched, and an efficient energy transfer from the PFO segments to the DBT units has occurred. The redshift of PL emission of the DBT units (shown by arrow) that are presented by the denser PFO-DBT nanorod bundles well correlated with the redshift of its UV-vis absorption. PFO emission has completely quenched and being dominant by the DBT emission. This phenomenon could be due to the incorporation of the DBT units into the PFO segments which hence leads to the better conjugation length and chain alignment produced by the PFO-DBT nanorod bundles.

## Conclusions

In the present study, the effect of different spin coating rates on the morphological, structural, and optical properties of PFO-DBT nanorod bundles is reported. Polymer solution has been demonstrated to have different characteristics and abilities to infiltrate into the cavities at different spin coating rates. Highly dense PFO-DBT nanorod bundles are obtained at low spin coating rate with enhancement of structural and optical properties.

## Abbreviations

FESEM: field emission scanning electron microscopy; FWHM: full-width half maximum; NaOH: sodium hydroxide; PFO-DBT: poly[2,7-(9,9-dioctylfluorene)-alt-4,7-bis(thiophen-2-yl)benzo-2,1,3-thiadiazole]; PL: photoluminescence; rpm: rotation per minute; TEM: transmission electron microscopy; XRD: X-ray diffraction.

## Competing interests

The authors declare that they have no competing interests.

## Authors' contributions

MSF carried out the experiment, participated in the sequence alignment, and drafted the manuscript. AS participated in the design of the study, performed the analysis, and helped draft the manuscript. KS conceived of the study and helped draft the manuscript. All authors read and approved the final manuscript.

## Authors' information

MSF is currently doing his Ph.D. at the University of Malaya. AS and KS are senior lecturers at the Department of Physics, University of Malaya. AS's and KS's research interests include the synthesis of nanostructured materials via template-assisted method and applications in organic electronic devices such as sensors and photovoltaic cells.
